# Precision and Safety of Ultrasound-Guided versus Palpation-Guided Needle Placement on the Patellar Tendon: A Cadaveric Study

**DOI:** 10.3390/life13102060

**Published:** 2023-10-15

**Authors:** José L. Arias-Buría, Sergio Borrella-Andrés, Jacobo Rodríguez-Sanz, Carlos López-de-Celis, Miguel Malo-Urriés, César Fernández-de-las-Peñas, Gracia M. Gallego-Sendarrubias, Vanessa González-Rueda, Albert Pérez-Bellmunt, Isabel Albarova-Corral

**Affiliations:** 1 Department of Physical Therapy, Occupational Therapy, Rehabilitation and Physical Medicine, Universidad Rey Juan Carlos. Av. de Atenas, s/n, Alcorcón, 28922 Madrid, Spain; 2 Health Sciences Faculty, Department of Physiatry and Nursing, University of Zaragoza, C/Domingo Miral S/N, 50009 Zaragoza, Spain; 3 Faculty of Medicine and Health Science, Universitat Internacional de Catalunya C/Josep Trueta S/N, Sant Cugat del Vallés, 08195 Barcelona, Spain; 4 ACTIUM Functional Anatomy Group, Sant Cugat del Vallés, 08195 Barcelona, Spain; 5 Fundació Institut Universitari per a la Recerca a l’Atenció Primària de Salut Jordi Gol i Gurina, 08007 Barcelona, Spain; 6 Department of Physical Therapy, Camilo José Cela University. C. Castillo de Alarcón, 49, Villafranca del Castillo, 28692 Madrid, Spain

**Keywords:** needle, ultrasound, patellar tendon, knee, accuracy, palpation

## Abstract

For decades, needling interventions have been performed based on manual palpation and anatomic knowledge. The increasing use of real-time ultrasonography in clinical practice has improved the accuracy and safety of needling techniques. Although currently ultrasound-guided procedures are routinely used for patellar tendon pathology, e.g., during percutaneous electrolysis, the accuracy of these procedures is still unknown. This study used a cadaveric model to compare and evaluate both the accuracy and safety of ultrasound-guided and palpation-guided needling techniques for the patellar tendon. A total of five physical therapists performed a series of 20 needle insertion task each (n = 100), 10 insertions based on manual palpation (n = 50) and 10 insertions guided with ultrasound (n = 50) to place a needle along the interface between the patellar tendon and Hoffa’s fat pad. All procedures were performed on cryopreserved knee specimens. Distance to the targeted tissue, time of the procedure, accurate rate of insertions, number of passes, and unintentional punctured structures between both applications (with and without ultrasound guiding) were compared. The results revealed higher accuracy (100% vs. 80%), a lower distance from needle to the targeted tissue (0.25 ± 0.65 vs. 2.5 ± 1.9 mm), longer surface of contact with the needle (15.5 ± 6.65 vs. 4.7 ± 7.5 mm), and a lower frequency of patellar tendon puncture (16% vs. 52%, *p* < 0.001) with the ultrasound-guided procedure as opposed to palpation-guided one. Nevertheless, the ultrasound-guided procedure took longer (54.8 ± 26.8 vs. 23.75 ± 15.4 s) and required more passes (2.55 ± 1.9 vs. 1.5 ± 0.95) to be conducted than the palpation-guided procedure (all, *p* < 0.001). According to these findings, the accuracy of invasive procedures applied on the patellar tendon is higher when conducted with ultrasound guidance than when conducted just on manual palpation or anatomical landmark. These results suggest that ultrasound could improve the clinical application of invasive procedures at the fat-patellar tendon interface. Due to the anatomical features of the targeted tissue, some procedures require this precision, so the use of ultrasound is recommended.

## 1. Introduction

Patellar tendinopathy or jumper’s knee is a pain condition highly prevalent among athletes in different sports and is characterized by anterior knee pain and tenderness on the patellar tendon. Approximately 6–22% of athletes may suffer from patellar tendinopathy at some point during their sport career [1,2] and can reach prevalence rates of 32–45% in specific sports such as volleyball and basketball [3]. Patellar tendinopathy usually limits activities of daily life and sport practice. More than one-third of athletes with this condition cannot return to regular sport practice for at least six months [4].

The patellar tendon receives high loads during functional activities, particularly with jumping exercises. When the tendon healing response fails, chronic repetitive tendon overload may result in microscopic failure with a decrease in mechanical properties leading to posterior tendinopathy [5]. This process is favored by the low metabolism and blood supply of the patellar tendon, as well as other factors including weight, leg length difference, quadriceps/hamstrings flexibility, or deficit in muscular strength [6]. The most common point of degeneration of the patellar tendon (targeted tissue) is located at the proximal (upper) and deep portion of the tendon [7] which may be due to the elevated mechanical stress this tendon area receives since it is closed to the knee rotation center [8] or due to an intrinsic impingement mechanism [9]. The interface between the patellar tendon and Hoffa’s fat pad has been also shown to be of clinical relevance for the development of pain symptoms, as well as being a key element in the regenerative process [10].

Conservative interventions are the first-line treatment for patellar tendinopathy. Several conservative treatments, e.g., non-steroidal anti-inflammatory drugs, exercises, manual therapy, extracorporeal shockwave therapy or injections are proposed for managing this condition [11,12,13]. Emerging techniques such as percutaneous electrolysis have been recently proposed for treating pain and function in individuals with patellar tendinopathy [14,15,16]. In fact, percutaneous electrolysis has been shown to reduce pain intensity and associated disability in musculoskeletal pain disorders, including patellar tendinopathy, based on moderate-quality evidence [17]. Ultrasound-guided percutaneous electrolysis applies a galvanic electrical current through a filiform solid needle placed in the targeted tissue (e.g., the patellar tendon). This technique causes an inflammatory response in the affected tissue and a significant rise in pH [basic] at the needle’s tip [18]. Hence, percutaneous electrolysis is used as an electrochemical procedure that can induce a regulated inflammatory response in the targeted tissue, facilitating phagocytosis of the degenerated tissue and enabling a subsequent site-specific repair process. [19]. An animal study found that the application of percutaneous electrolysis in an experimentally induced Achilles tendinopathy model is able to increase the expression of the different genes associated with collagen regeneration and remodeling of extracellular matrix [20]. 

Any invasive intervention such as percutaneous electrolysis requires an accurate needle placement. In fact, the American Society of Regional Anesthesia and Pain Medicine considers incorrect needle placement as a major factor in the reduction in clinical effectiveness and a risk for adverse effects with these interventions [21]. For needling interventions, needle placement is clinically performed based on manual palpation and anatomical knowledge. Anatomical landmarks represent the most common clinical method for applying some invasive interventions, e.g., trigger point dry needling. Nevertheless, the safety of needling interventions based on anatomical palpation is sometimes questioned due to the development of adverse events [22].

The development and growing use of imaging procedures and guidance methods have been implemented to improve needling interventions accuracy and to reduce the adverse effects of these approaches. Ultrasound provides real-time guidance that facilitates an accurate placement of the needle and minimize the incidence of unintentional puncture of sensitive surrounding tissues [23,24,25]. Thus, ultrasound-guidance has become a standard for some techniques such as percutaneous electrolysis. In fact, most studies investigating the clinical effectiveness of percutaneous electrolysis applied the intervention ultrasound-guided. However, no scientific evidence exists to date supporting that needling insertion with the guide of ultrasound imaging is more accurate and/or safe than needling insertion based on manual palpation and anatomical landmarks. Ultrasound guidance can be used for increasing the accuracy and safety of procedures targeting deep structures or interface areas, e.g., the interface between the patellar tendon and the Hoffa’s fat pad. The objective of this study was to compare the ultrasound-guided procedure to the palpation-guided procedure in terms of accuracy, performance time, number of passes or incidence of unintentional puncture of major structures when targeting the patellar tendon. We hypothesized that the use of ultrasound guidance will increase accuracy of the needling insertion into the targeted tissue, in the current study, the interface between the patellar tendon and the Hoffa’s fat pad.

## 2. Methods

### 2.1. Participants

This study was performed at the Department of Basic Sciences of Universitat Internacional de Catalunya (Barcelona, Spain). The Ethics Committee (CBAS-2021-09; Comitè d’Ética de Recerca, International University of Catalonia) evaluated the protocol and approved the study. This article adheres to the applicable Enhancing the Quality and Transparency of Health Research (EQUATOR) network guidelines. A validation study comparing palpation-guided versus ultrasound-guided techniques was designed and performed in 3 phases, as follows: (a) preparation of the cadaveric model, (b) 10 min theoretical audio-visual presentation of the objectives of the study and practice tasks, and (c) hands-on evaluation of the procedure. A total of five physical therapists specialized in musculoskeletal needling interventions with more than 10 years of experience participated in the current study.

In this study, cryopreserved cadaver specimens provided by donors to the institutional university anatomy laboratory of Universitat Internacional de Catalunya (Barcelona, Spain) were utilized. To preserve tissue characteristics, the frozen samples were preserved at −20 °C and thawed 48 h before the procedure at room temperature. All bodies were frozen within less than 24 h after death. Therefore, only one thawing cycle was performed with the cadavers. According to current literature, one-time freezing of human tissues is practically harmless for the integrity of the respective tissue [26,27]. Repeated freeze-thawing (more than 5 times), however, leads to a decrease in integrity with the increasing number of freeze-thaw cycles. The cadavers were placed in a similar position to which a patient is clinically placed, with an optimized ergonomics, including the handling of the transducer and needle insertions, mimicking common clinical practice.

All therapists underwent a standardized 10 min instructional and practical session to understand the aim of the study and to become familiar with the procedure.

### 2.2. Procedure

**Figure 1:**
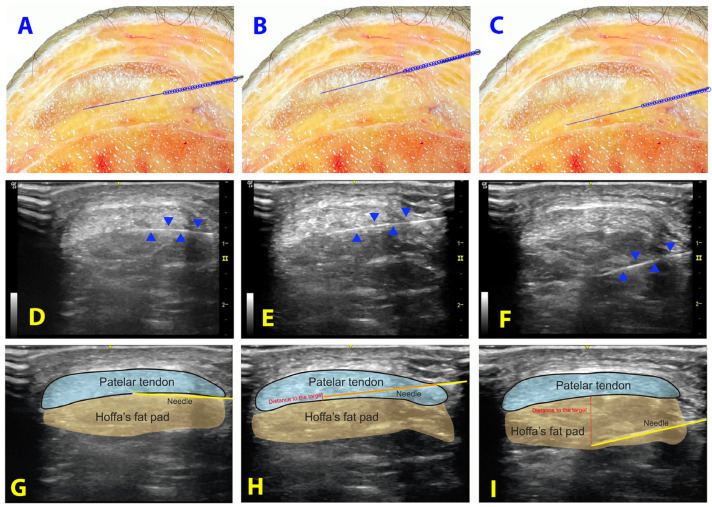
Cadaveric and ultrasonography scheme of the procedure of the interface between the patellar tendon and Hoffa’s fat pad in the cadaver (**A**–**C**) and ultrasound-guided (**D**–**I**). Proper positioning on the cadaver (**A**) and ultrasound-guided (**D**–**G**). Positioning inside the patellar tendon on the cadaver (**B**) and ultrasound-guided (**E**–**H**); the distance between the needle’s tip and the interface is shown in red; the distance of patellar tendon punctured is shown in orange. Positioning of the needle away from the interface on the cadaver (**C**) and ultrasound-guided (**F**–**I**); the distance between the needle’s tip and the interface is shown in red.

Therapists were asked to place the needle at the interface (targeted tissue) between the patellar tendon and Hoffa’s fat pad of the cadaver. Contact with this interface should be done for as long as possible, avoiding entering into the tendon (target-contact task, Figure 1A). Therapists performed as many needling passes as they considered necessary until final accurate placement was perceived [28]. This procedure was conducted under the two conditions considered in this study, anatomical landmarks/palpation-guided and ultrasound-guided. Each participant/therapist completed a total of 20 needle tasks (10 insertions based on manual palpation and 10 insertions guided with ultrasound imaging) with a 5 min rest after 10 attempts to prevent fatigue [29,30].

**Figure 2:**
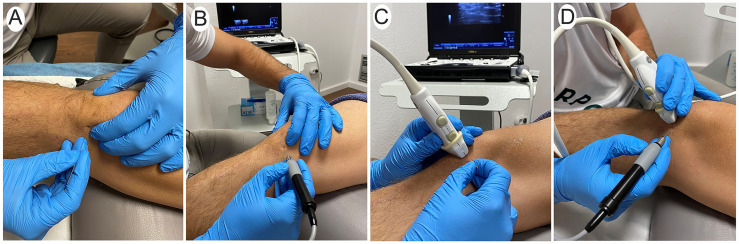
Procedure of the invasive techniques guided by palpation (**A**,**B**) and guided by ultrasound imaging (**C**,**D**). The technique is performed without handpiece (**A**,**C**) and with a percutaneous electrolysis handpiece (**B**,**D**).

Condition A (Palpation-guided procedure): Participants/therapists were asked for conducting the needling insertion first with the guidance of their palpatory skills according to anatomical landmarks. The inferior pole of the patella and the medial and lateral limits of the patellar tendon were identified and used as the anatomical landmarks for the insertion. The dominant hand inserted the needle from the medial side of the patella into the proximal portion of the interface between the Hoffa’s fat pad and patellar tendon (Figure 2A,B). First, each therapist performed 10 palpation-guided attempts without the use of the ultrasound to avoid the pre-visualization of the ultrasound that could help palpation-guided approaches. From the 10 palpation-guided attempts, 5 were conducted without the use of the handpiece (mimicking the application of tendon dry needling treatment, Figure 2A) and the remaining five were conducted with the use of the handpiece (mimicking the application of percutaneous electrolysis intervention, Figure 2B).

Condition B (Ultrasound-guided procedure): Participants/therapists were asked to complete the needling insertion under ultrasound imaging guidance. A LOGIQ e R8 (General Electric Healthcare) ultrasound equipment with a 4 to 12 MHz linear transducer was used. Therapists followed a standardized protocol to pre-calibrate and optimize the ultrasonographic image parameters, including frequency, depth, gain, and focus. This was done to ensure that participants could focus on the technique. Participants/therapists were instructed to perform the procedure by using an “in-plane” imaging approach, i.e., in a transversal view of the patellar tendon on the ultrasound. The probe, held in the non-dominant hand, was initially positioned in the sagittal plane of the knee to identify the most proximal region of the patellar tendon. The probe was then rotated 90° into a transverse plane, to obtain an optimal view of the interface between the patellar tendon and Hoffa’s fat pad. Once an optimal visualization of these structures was identified in the ultrasound screen, the needle was inserted with the dominant hand from the medial side using the same procedure than in condition A (Figure 2C,D). The “in-plane” ultrasound imaging approach permits one to visualize the trajectory of the needle and proper control of the needle trajectory in a real-time ultrasound (Figure 1D–F).

All insertions were performed with sterile stainless-steel solid filiform needles (0.30 × 40 mm, Agupunt, Barcelona). The needle was inserted directly in condition A (5 attempts based on anatomical landmarks and 5 attempts with ultrasound guiding, Figure 2A,C) and with the needle inserted into a handpiece of percutaneous electrolysis equipment in condition B (5 attempts based on anatomical landmarks and 5 attempts with ultrasound guiding, Figure 2B,D). The insertion attempts using the handpiece were randomly selected following a computerized random assignment list.

### 2.3. Measurements

Following each needle placement, two external researchers with more than 10 years of experience registered the following measures in a blinded design (Figure 1G–I):

- Distance: Distance of the needle’s tip to the target (in millimeters).
- Target contact and surface of contact: Contact of the needle with the interface between patellar tendon and Hoffa’s fat pad (yes/no) and the distance of contact (in millimeters).
- Punctured structure: If a different structure was punctured (patellar tendon or cortical bone). If the patellar tendon and not the interface was punctured, the distance from the needle to the interface between patellar tendon and Hoffa’s fat pad was recorded (in millimeters).
- Time: The time needed for a single needle insertion (in seconds).
- Passes: The number of needle insertion (each time the participant/therapist advanced the needle after a change of direction was considered one pass) [28].
- Needle length outside: The length of the needle that was located outside the body (in millimeters).
- Accuracy: The inserted attempt was considered accurate if the tip of the needle was properly placed less than 3 mm from the target or the contact with the interface was properly achieved.

### 2.4. Statistical Analysis

Data were analyzed with the IBM SPSS™ statistics 22.0 software. Descriptive statistics were used to report the data, including total counts, percentages, means, and their respective standard deviations (SD). The normality of the variables was assessed through the Kolmogorov–Smirnov test. Comparative analyses of the quantitative measurements (distance to the target, distance to the patellar tendon, time required for the procedure, number of passes, needle length outside) between palpation-guided and ultrasound-guided procedures and between use of not of the handpiece were performed using independent student *t*-tests. The chi-square (χ^2^) test was used to determine the differences in nominal variables (contact with the targeted tissue, puncture of the patellar tendon, and success/failure of the procedure). The significance level was set at 0.05.

## 3. Results

Table 1 summarizes clinical characteristics of the participants/therapists involved in the study and overall data of the procedures.

The ultrasound-guided procedure significantly increased the number of contacts (50 vs. 26, *X*^2^ = 31.579, *p* < 0.001) and the surface of needle contact with the targeted tissue (15.5 ± 6.65 mm vs. 4.7 ± 7.5 mm, *t* = −7.694, *p* < 0.001) when compared with the palpation-guided procedure. The accuracy rate of the procedure improved significantly from 80% with palpation-guided to 100% when ultrasound-guided (*X*^2^ = 11.112, *p* < 0.001). Further, the ultrasound-guided procedure was associated with a significant (*X*^2^ = 14.439, *p* < 0.001) lower frequency of patellar tendon puncture (16%, 8/50) when compared with palpation-guided procedure (52%, 26/50). Finally, when the procedure was ultrasound-guided, a lower distance from the tip of the needle to the target (0.25 ± 0.65 mm) was also observed when compared with a palpation-guided procedure (2.5 ± 1.9 mm, *t* = 7.938, *p* < 0.001). Nevertheless, using ultrasound guide required more time (54.8 ± 26.8 vs. 23.75 ± 15.4 s, *t* = −7.102, *p* < 0.001) and more needle passes (2.55 ± 1.9 vs. 1.5 ± 0.95, *t* = −3.581, *p* < 0.001) than when doing the procedure with manual palpation. Table 2 details the measurements with both palpation-guided and ultrasound-guided procedures.

The comparison of the measurements between the use or non-use of the handpiece did not reveal any significant difference between both procedures (Table 3)

## 4. Discussion

This cadaveric study found that ultrasound-guided needle insertion significantly increased the accuracy of the procedure while reducing the incidence of unintentional punctures of the patellar tendon compared to needle insertion based on manual palpation. The accuracy of the insertion when ultrasound-guided was 100% whereas the accuracy of the insertion based on anatomical landmarks was 80%. However, performing the procedure guided by ultrasound required significantly more time and more needle passes than performing the procedure manually.

Ultrasound-guided needle precise placement has become a universal skill required in several healthcare professions. Multiple interventional procedures performed by different health professionals, including percutaneous electrolysis, require the insertion of a needle into the human body, thus, accurate placement of the needle into the targeted tissue is a requirement of any of these interventions [15,16,31,32,33]. Therefore, it appears that inaccurate needle placement is the primary factor contributing to a decrease in the effectiveness of needling interventions and represents the primary risk factor for adverse effects [21]. Several minimally invasive techniques are clinically conducted with the assistance of palpation; however, with the development of imaging techniques, new procedures have increased their accuracy and safety. Ultrasound provides real-time visual guidance that could increase the accuracy and reduce the adverse effects of minimally invasive techniques [21,34,35]. Nevertheless, despite being used routinely in clinical practice, the precision and other parameters of these techniques is still unknown.

Our study results demonstrated a significant reduction in the distance between the needle and the targeted tissue, e.g., interface between patellar tendon and Hoffa’s fat pad, when employing ultrasound guidance. This finding supports the notion of precise needle tip placement, as previously suggested [23,24,25]. Previous studies with different ultrasound guidance procedures have obtained accuracy rates between 1.5 mm and 3.27 mm when using phantoms [28,36,37]. Our study aimed to achieve a target–contact task, i.e., the contact with the interface should be as long as possible with the needle for avoiding entering into different surrounding tissues such as the tendon. Therefore, whether a contact was reached and the millimeters of that contact was also measured. The ultrasound-guided procedure significantly increased the number of accurate contacts and the surface of contact with the interface. To the best of the authors knowledge, no anatomical study has investigated if a solid needle can accurately and safely reach the interface between the patellar tendon and Hoffa’s fat pad guided by palpation or ultrasound. Regarding the safety of the technique, the ultrasound-based guidance was also associated with a lower frequency of patellar tendon puncture (16% vs. 52%). In agreement with our results, previous studies of regional anesthesia have also demonstrated greater safety of needling intervention with ultrasound-guided procedures [21].

Nevertheless, we also observed that to achieve this accuracy and safety, ultrasound-guided procedure required significantly more time and more needle passes than the palpation-guided procedure. Contrary to our results, previous studies found that ultrasound guidance can reduce the time of the procedure [25,29,38] and the number of passes [39]. These differences may be due to the fact that the technique evaluated in the current study, unlike previous studies, is a target–contact task, which requires a longer time to achieve a larger and accurate contact surface and, therefore, needs higher precision. In any case, although ultrasound-guided procedure would require significantly more time and more passes, the difference was approx. 30 s longer and an additional pass of the needle, which is of small clinical significance.

Ultrasound imaging is a simple, portable, inexpensive, non-ionising, accessible, and well-tolerated tool to guide invasive procedures of several medical interventions, such as nerve blocks in anesthesia, injections of musculoskeletal structures, central venous access, thoracocentesis, paracentesis, amniocentesis, or interventional radiology such as liver or renal biopsies [40]. Other healthcare professions such as physiotherapy also use ultrasound guidance for invasive techniques such as dry needling, electrical nerve stimulation (neuromodulation), or percutaneous electrolysis. Ultrasound guidance of invasive techniques can be performed in different ways. First, a pre-procedural ultrasound scan without guidance during the intervention is a traditional approach that can be used to determine the point of insertion, direction and depth of the needle. Second, real-time ultrasound guidance maintains the image of the structure and needle during the invasive technique. These real-time techniques can be performed with the ultrasound beam parallel or perpendicular to the needle, referred to as “in plane” or “out of plane”, respectively. The “in plane” techniques such as those used in this study, are easier to learn and perform, and have the advantage of enabling the professional to follow the needle shaft and tip directed toward the target during the intervention. However, it may result in a false sense of security [41,42,43].

The targeted tissue was the interface between patellar tendon and Hoffa’s fat pad in the proximal insertion of the patellar tendon. This is an area of high clinical relevance as it frequently presents structural alterations [15]. Current treatments of patellar tendinopathy try to address the peripheral area of the tendon [15] instead of targeting intra-tendinous tissue. From a therapeutic point of view, the periphery of the tendon is an important area, as it accumulates algogenic substances with neovascularization and neoinnervation processes [44]. There is evidence indicating that the application of percutaneous electrolysis targeting the peripheral tendon improved pain and functionality of patellar tendon [15,16]. Percutaneous electrolysis is a pioneering technique that produces an electrochemical ablation using galvanic current directly applied to the clinical point of degeneration, promoting a regenerative reaction. Several authors have proposed that percutaneous electrolysis combine mechanical and electro—chemical effects to produce several physiological changes including a reduction in pro-inflammatory mediators (TNF-α, IL-1β, IL-6, CCL3, CCL4, CCL5, CCR8, NF-κB), an increase in the expression of anti-inflammatory proteins (PPAR-γ, IL10, IL13), as well as an increase in vascular growth factor (VEGF) and its receptor (VEGF-R1) [18,45]. The results of the current study should be considered by practitioners performing percutaneous electrolysis or other needing interventions. Ultrasound-guided techniques enable practitioners to place the needle more precisely to the targeted tissue and reduce the incidence of unintentional puncture of surrounding tissues. This can be especially important in anatomical areas of risk, which will require more accuracy.

Finally, we should recognize some limitations of this study that should be considered when interpreting the findings. First, the fact that the procedure was always first manually conducted before using ultrasound guide may have induced a potential bias due to fatigue of the therapist in the latter tasks. To avoid this possible fatigue of the therapist, rest times after each needle insertion and after each block of 10 attempts were used. The reason for starting with the palpation-guided procedure was the possibility of a learning bias if the ultrasound-guided procedure had been done first. This is based on the fact that the visual feedback of ultrasound imaging has demonstrated to be a crucial factor for the learning process [46]. Second, techniques were applied by five therapists with experience in ultrasound procedures. Future studies investigating intra- and inter-operator reliability and differences between therapists with different levels of training and experience are needed. Third, we used a human cadaveric design. Previous studies have been mostly performed on synthetic phantoms or animal cadaveric samples [28,30,36,37,47,48]. In fact, authors of these studies consider that further research in humans rather than in animals are necessary [28]. Our study covers this proposal since the human cadaver is more realistic in anatomical terms and could better mimic clinical practice than animal models. However, current results must be interpreted with caution considering that a cadaver sample is not the same as a human (in vivo) sample. Future studies should investigate the accuracy of the needle techniques applied in other areas than the patellar tendon, as well as in other tissues, e.g., nerve trunks. Finally, although ultrasonographic guidance is increasingly popular among professionals who treat musculoskeletal conditions, several doubts exist about the economic costs for using this technology for simple invasive techniques. Our study did not address the associated economic costs, including acquisition and maintenance of the ultrasound equipment and it consumables. Thus, cost–benefit studies are required to determine the economic implications of sonographic guidance for invasive procedures.

## 5. Conclusions

Although needle insertion based on manual palpation or anatomical landmarks exhibits an acceptable level of precision, the use of an ultrasound guide significantly improved the accuracy (distance to the target, target contact, and surface of contact) and safety (lower frequency of unintentional patellar tendon puncture) of the procedure. However, the use of ultrasound imaging required more time and more passes. Future studies in humans are needed to further confirm these results and to identify if clinical differences exists based on each approach.

## Figures and Tables

**Table 1:** Clinical characteristics of the participants/therapists and overall data of the procedures.

	Mean (SD)
Male/Female (n)	4/1
Experience with invasive techniques (years)	10.0 ± 5.4
Experience with ultrasound (years)	8.8 ± 3.5
Total needle procedures (n)	100
Palpation-guided/Ultrasound-guided (n)	50/50
With/without handpiece (n)	50/50

Abbreviations: n, number; SD, standard deviation.

**Table 2:** Measurements with palpation-guided and ultrasound-guided interventions.

	Palpation-Guided	Ultrasound-Guided	
	Mean (SD)	Mean (SD)	*p*
Distance to the target (mm)	2.5 (1.9)	0.25 (0.65)	<0.001
Target contact (no/yes)	24/26	0/50	<0.001
Surface of contact with the target (mm)	4.7 (7.5)	15.5 (6.65)	<0.001
Patellar tendon punctured (no/yes)	24/26	42/8	<0.001
Distance of patellar tendon punctured (mm)	2.95 (6.25)	2.3 (12.8)	0.739
Success/Failure (n)	40/10	50/0	0.001
Time required (s)	23.75 (15.4)	54.8 (26.8)	<0.001
Passes (total number)	1.5 (0.95)	2.55 (1.9)	0.001
Needle length outside (mm)	9.25 (5.95)	6.0 (4.25)	0.003

Abbreviations: n, number; mm, millimeters; s: seconds; SD, standard deviation.

**Table 3:** Measurements comparing the use or not of percutaneous electrolysis handpiece.

	With Handpiece	Needle	
	Mean (SD)	Mean (SD)	*p*
Distance to the target (mm)	1.4 (1.8)	1.3 (1.8)	0.875
Target contact (no/yes)	11/39	13/37	0.812
Surface of contact with the target (mm)	9.9 (8.6)	10.3 (9.3)	0.789
Patellar tendon punctured (no/yes)	29/21	37/13	0.142
Distance of patellar tendon punctured (mm)	3.9 (13.4)	1.4 (4.6)	0.216
Success/Failure (n)	45/5	45/5	1.000
Time required (s)	39.6 (30.0)	39.0 (23.4)	0.901
Passes (total number)	1.9 (1.5)	2.1 (1.7)	0.534
Needle length outside (mm)	6.85 (5.1)	8.5 (5.65)	0.134

Abbreviations: n, number; mm, millimeters; s: seconds; SD, standard deviation.

## Data Availability

The data presented in this study are available on request from the corresponding author.
